# CCR6 Is a Prognostic Marker for Overall Survival in Patients with Colorectal Cancer, and Its Overexpression Enhances Metastasis *In Vivo*

**DOI:** 10.1371/journal.pone.0101137

**Published:** 2014-06-30

**Authors:** Jinlin Liu, Fang Ke, Zhenyao Xu, Zhaoyuan Liu, Lingyun Zhang, Sha Yan, Zhe Wang, Hong Wang, Honglin Wang

**Affiliations:** 1 Shanghai Institute of Immunology, Institute of Medical Sciences, Shanghai Jiao Tong University School of Medicine, Shanghai, China; 2 Shanghai Key Laboratory for Tumor Microenvironment and Inflammation, Shanghai Jiao Tong University School of Medicine, Shanghai, China; Sun Yat-sen University Medical School, China

## Abstract

The chemokine receptor CCR6 has been recently shown to be associated with colorectal cancer (CRC) progression. However, the direct evidence for whether CCR6 in tumors is a prognostic marker for the survival of patients with CRC and whether it plays a critical role in CRC metastasis *in vivo* is lacking. Here we show that the levels of CCR6 were upregulated in CRC cell lines and primary CRC clinical samples. CCR6 upregulation was closely correlated with disease stages and the survival time of CRC patients. Knockdown of CCR6 inhibited the migration of CRC cells *in vitro*. Overexpression of CCR6 in CRC cells increased their proliferation, migration, and colony formation *in vitro* and promoted their metastatic potential *in vivo*. CCR6 activated Akt signaling, upregulated metastasis genes and downregulated metastasis suppressor genes. Selective targeting of CCR6 in tumors dramatically inhibited the growth of CRC in mice. Thus, the tumor expression of CCR6 plays a critical role in CRC metastasis, upregulated CCR6 predicts poor survival in CRC patients, and targeting CCR6 expression in tumors may be a potential therapeutic strategy for CRC.

## Introduction

Colorectal cancer (CRC) is a major health concern worldwide. Although substantial progress has been made in the past decade, the challenges of treating CRC and its metastases remain formidable [1]. Currently, despite the use of specific active drugs for the treatment of metastatic CRC becoming more popular, cure rates are low and the underlying molecular mechanisms for the organ-oriented metastasis of CRC are not fully understood.

Chemokines are 8- to 12-kDa peptides that function as chemoattractant cytokines and exert their biological effects by interacting with G protein-linked transmembrane chemokine receptors. A number of chemokines and their corresponding receptors are known to play an important role in leukocyte trafficking and homing, especially at sites of inflammation, tissue damage and malignant cell migration [2], [3]. Interestingly, while most chemokine receptors bind to multiple chemokines, the chemokine receptor CCR6 has only one chemokine ligand, CCL20 (previously known as macrophage inflammatory protein-3α or MIP-3α) [4].

CCR6 is primarily expressed on leukocytes, with expression in mature lymphocytes, especially in memory cells [4], immature dendritic cells (DCs) of particular lineages [5] and migrating regulatory T cells [6]. In most cases, CCR6 is absent from granulocytes (except activated neutrophils), monocytic cells, immature lymphocytes, and mature DCs [7]–[9]. CCL20 shows both constitutive and inducible expression, mainly in mucosa-associated lymphoid tissues and the liver [10], and the basal expression rate is increased under inflammatory conditions [11]. The basal expression level of CCL20 is thought to regulate the migration of CCR6-expressing immature DCs and memory lymphocytes from the blood for homeostatic surveillance. The upregulation of CCL20 during inflammation may enhance the migration of both of these cell types into the tissue [12]–[14]. For human cancers, accumulated data imply an association between the chemokine-chemokine receptor system and the metastatic potential of cancer cells. For example, tumor cells from at least 23 different types of human cancers of epithelial, mesenchymal and hematopoietic origin express CXCR4 [15], [16]. CCR7 was also found in breast, gastric, and esophageal squamous cancer, and its expression was correlated with poor prognosis [15], [17], [18]. All these studies show that the expression of chemokine receptors in cancer metastasis is not random.

The chemokine receptor CCR6 is of particular interest in the liver metastasis of colorectal cancer. Its unique chemokine ligand CCL20 is predominantly expressed in lymphatic tissue and in the liver [19]. The aberrant expression of the chemokine receptor CCR6 on CRC cells is reportedly involved in organ-selective tumor metastasis [20], [21]. However, the direct *in vivo* evidence supporting a role for CCR6 in the metastasis of CRC is lacking. In the present study we found that upregulated CCR6 expression in metastatic CRC cell lines predicted poor survival for CRC patients. The overexpression of CCR6 was sufficient to promote CRC cell metastasis both *in vitro* and *in vivo*. Selectively blocking the CCR6 function dramatically inhibited the growth of CRC in mice. CCR6 exhibits a direct role in the metastasis of human CRC, possibly by regulating metastasis-related genes.

## Materials and Methods

### Ethics Statement

This study was approved by the Ethics Committees of Hospital of Shanghai Jiao Tong University School of Medicine, China.

All mice were kept under specific-pathogen-free (SPF) conditions in compliance with the National Institutes of Health Guide for the Care and Use of Laboratory Animals and with the approval (SYXK-2003-0026) of the Scientific Investigation Board of the Shanghai Jiao Tong University School of Medicine, Shanghai, China. To ameliorate any suffering of mice observed during these experimental studies, mice were euthanized by CO_2_ inhalation.

A tissue microarray including 191 human colorectal cancer and corresponding para-tumor samples was purchased from the National Engineering Center for biochips at Shanghai. Written informed consent from the donor was obtained for use of their samples for research purposes. None of the patients received chemotherapy or radiotherapy prior to surgical resection. The adjacent non-cancerous colonic tissues were obtained from a minimum of 2 cm away from the tumor to ensure that these tissues were free of cancerous cells. Specimens were routinely fixed in 10% formalin in the immediate postoperative period and embedded in paraffin within 24 hrs of removal.

### Mice

C57BL/6J, Balb/c and B6.129P2-Ccr6tmlDgen/J (designated as CCR6^−/−^) mice were purchased from the Jackson Laboratory (Bar Harbor, ME).

### CRC Cell lines and Cell Cultures

An immortalized human embryonic kidney cell line, HEK293T, was cultured in Dulbecco’s Modified Eagle’s Medium (DMEM, Hyclone) with 10% fetal bovine serum (FBS). The mouse colorectal cell line CMT93 was maintained in DMEM supplemented with 10% FBS. Another mouse colorectal cell line, CT26, was maintained in RPMI-1640 medium with 10% FBS. HEK293T, CMT93, CT26 and Seven human CRC cell lines, Caco-2, SW480, HT-29, HCT116, SW1116, SW620 and LoVo, were all obtained from the Cell Bank of the Committee on Type Culture Collection of the Chinese Academy of Sciences (Shanghai, China), where they were characterized by DNA fingerprinting, mycoplasma detection, and cell vitality. Caco-2 cells were grown in Eagle's Minimum Essential Medium (Gibco) with 20% FBS. HT-29 and HCT116 cells were grown in McCoy's 5a Medium (Gibco) with 10% FBS. SW1116, SW480 and SW620 cells were grown in Leibovitz's L-15 Medium (Gibco) with 10% FBS. LoVo were grown in F-12K Medium (Gibco) with 10% FBS. All cells were grown in a humidified atmosphere of 5% CO_2_ and 95% air except SW1116, SW480 and SW620, which were grown in 100% air at 37°C.

### Immunohistochemistry

For immunohischemistry, tissue sections (5**µm) were cut from routine blocks, de-paraffinized with xylene, rehydrated, and subjected to heat-induced epitope retrieval (HIER) methods before incubation with the appropriate antibodies. Sections were immersed in 10**mM sodium citrate buffer at pH 6.0 and were subsequently heated in a pressure cooker for 10**min. After rinsing in running water and PBS, the sections were incubated in TBST/5% normal goat serum blocking solution for 0.5 hr at room temperature and then incubated overnight at 4°C with a monoclonal anti-human CCR6 antibody (1∶50 dilution; R&D System, clone: #53103). This dilution was considered optimal after antibody titration using human tonsil as a positive control. An irrelevant mouse IgG_2b_ antibody was used as an isotype control in all cases to demonstrate that staining was specific for CCR6. On the next day, sections were labeled with an appropriate HRP-conjugated secondary antibody, developed with diaminobenzaminidine (DAB) and counterstained with hematoxylin.

### Evaluation of Immunohischemistry Staining

For visual assessment, the assessment of immunostaining was performed independently by two pathologists who were blinded to clinical data of patients. As previously performed by others [18], we created an immunoreactive score by multiplying the score for the percent of positive cells, and the score for staining intensity. The score for the percentage of positive tumor cells was graded as follows: 0, none; 1, 1–24%; 2, 25–49%; 3, 50–74%; and 4, 75–100%. Immunostaining intensity was rated as follows: 0, none; 1, weak; 2, intermediate; and 3, intense. We divided all specimens (n = 191) into two groups (low or high CCR6 expression) according to the median principle. To compare the differential CCR6 expression in the cancerous colonic tissues and matched normal adjacent tissues in each clinical stage, the quantification of the specific CCR6 immunostaining intensity was performed using image gel software (IPWIN60). Briefly, the CCR6 immunostaining of all cancerous colonic tissues and matched adjacent non-cancerous colonic tissues on the tissue array chip were photographed with a microscope at 100-fold magnification (Carl Zeiss). The image gel software (IPWIN60) was used to calculate the OD values in captured pictures. The median OD value of three pictures from each core on the tissue array slides was used, and the differential CCR6 expression in the tumor and matched normal adjacent tissues at each clinical stage were compared.

### Wound Healing Assay

HCT116^Ctr^, HCT116^CCR6^, Caco-2^Ctr^, Caco-2^CCR6^, SW1116^Ctr^, SW1116^CCR6^, SW480^shCtr^, SW480^shCCR6^, LoVo^shCtr^ and LoVo^shCCR6^ cells were separately seeded into six-well plates, cultured until nearly 80% confluent, and serum starved for 24**hrs. Artificial wounds were created by scraping the monolayers with a sterile 10* µ*l tip, and the cells were washed with PBS several times to remove floating cells. Representative images of cells migrating into the wounds were captured after 0 hr and 24 hrs under a microscope (20×).

### Transwell Migration Assay

Transwell chambers consisting of 8-µm pore size membrane filter inserts (Corning, USA) were used to determine cell migration ability. FBS was used as the chemoattractant. Briefly, HCT116^Ctr^, HCT116^CCR6^, Caco-2^Ctr^, Caco-2^CCR6^, SW1116^Ctr^, SW1116^CCR6^, SW480^shCtr^, SW480^shCCR6^, LoVo^shCtr^ and LoVo^shCCR6^ cells were harvested and re-suspended in serum-free media. A quantity of 3×10^4^ cells was added into the upper chamber and incubated for 24**hrs at 37°C. Cells that had migrated through the membrane to the lower surface were fixed with cold methanol for 10**min, stained with 0.1% crystal violet for 30**min, washed, air dried, photographed and accounted.

### Colony Formation Assay

HCT116^Ctr^, HCT116^CCR6^, Caco-2^Ctr^, Caco-2^CCR6^, SW1116^Ctr^ and SW1116^CCR6^ cells were separately seeded into six-well plates at a density of 600 cells/well. The media were changed twice a week, and after 14 days, cells were fixed with methanol for 10 min, stained with 0.5% crystal violet for 15 min, rinsed three times with PBS to remove excess dye, photographed and counted.

### Western Blot Analysis

The following primary antibodies were used for western blotting: human CCR6 (#14-1969, eBioscience™ San Diego, USA), mouse CCR6 (#ab78429, Abcam), β-Actin (CP01, Calbiochem), Akt (pan) (C67E7) Rabbit mAb (#4691, Cell Signaling technology), phospho-Akt (Ser473) Rabbit mAb (#4060, Cell Signaling technology), phospho-Akt (Thr308) (C31E5E) Rabbit mAb (#2965, Cell Signaling technology), p44/42 MAPK (Erk1/2) (137F5) Rabbit mAb (#4695, Cell Signaling technology), phospho-p44/42 MAPK (Erk1/2) (Thr202/Tyr204) (20G11) Rabbit mAb (#4376, Cell Signaling technology). FXYD5 (#AP14909c, Abgent), SYK (#626201, Biolegend), uPAR (#sc-10815, Santa Cruz Biotechnology), CDH1 (#3195P, Cell Signaling technology), KISS-1 (# sc-18134, Santa Cruz Biotechnology), and TIMP2 (#635401, Biolegend). Western blot analysis was performed as previously published [22].

### Vector Construction

The lentiviral vector pGC-FU-Luc was constructed by introducing the firefly luciferase gene (Luc) downstream of the CMV promoter in the plasmid vector pGC-FU carrying the neomycin resistance gene. The lentiviral shuttle vector pLVX-IRES-ZsGreen1-CCR6 was constructed to stably overexpress CCR6 in Luc-HCT116. Briefly, the 1125-bp coding sequence of human CCR6 (NM_004367) was amplified from the cDNA template of human PBMCs and subcloned into the XhoI and BamHI sites of the pLVX-IRES-ZsGreen1 vector (Clontech Laboratories, USA). The lentiviral shuttle vector pLVXshRNA2 (Clontech Laboratories, USA) was used to express shRNAs from the U6 promoter. In addition to expressing shRNAs, pLVX-shRNA2 also expresses the green fluorescent protein ZsGreen1. For the knockdown of CCR6, four target sequences were designed. The target sequences used were as following: shCCR6-1, 5′-TCGACTCCAGTGAAGATTATT-3′; shCCR6-2, 5′-GGTCTATGACAGACGTCTATC-3′; shCCR6-3, 5′- TTTGTAGCTCTAGGGTATATA-3′; shCCR6-4, 5′- GACCAGTGAGACCGCAGATAA-3′. Scrambled control, 5′- TGTTCGCATTATCCGAACCAT-3′. Briefly, the complementary DNA oligonucleotides that consisted of a sequence-specific 21 nucleotide followed by the loop sequence (CTCGAG) and finally the reverse complement of the targeting sequence were synthesized, annealed, and inserted in pLVX-shRNA2 vector (Clontech) between BamHI and EcoRI sites downstream of the U6 promoter. Four pLVX-shRNA2-CCR6 shuttle plasmid were transient transfect into the HEK 293 T-cells with lipofectine, RT-PCR were screened for the most efficient CCR6 mRNA expression knockdown, one ShCCR6-1 plasmid showing nearly 75% decreased CCR6 mRNA in the SW480 cells were pick up for the following stable CCR6 knockdown CRC cell line construction.

### Lentivirus Production and Transduction

Lentiviruses were produced and harvested 72 hrs after transfection of 293 T cells with lentiviruses and the packaging plasmids pMD2.G and psPAX2 using ProFection Mammalian Transfection System (Promega). After filtering through a 0.45-µm low protein binding-polysulfonic filter (Millipore), the supernatant was collected separately and centrifuged for 10 min at 2000×g, 4°C before filtering through a 0.45-µm pore-size filter again. The flow-through was used directly for the transduction of human colorectal cancer cells. For the knockdown experiment, six clonal lines were screened for CCR6 expression by western blotting. Stably transfected SW480 and LoVo cell lines showing efficiently decreased CCR6 protein were screened and further purified by fluorescence activated cell sorting for downstream experiments. For the overexpression experiment, two transduced HCT116 cell lines were generated: HCT116 overexpressing ZsGreen1 (HCT116^Ctr^) and HCT116 overexpressing the bicistronic CCR6-ZsGreen1 genes (HCT116^CCR6^). The ZsGreen1^+^ HCT116 cells or ZsGreen1^+^ CCR6^+^ HCT116 cells were further purified by fluorescence activated cell sorting and expanded in the culture media. Similarly, Caco-2^Ctr^, Caco-2^CCR6^, SW1116^Ctr^ and SW1116^CCR6^ cells were also generated. In addition, three transduced HCT116 cell lines were produced for *in vivo* experiments: HCT116 stably overexpressing the Luc gene (Luc-HCT116) with G418 selection, HCT116 with overexpressing the Luc gene and the ZsGreen1 gene (Luc-HCT116^Ctr^), and HCT116 with overexpressing the Luc gene and the bicistronic CCR6-ZsGreen1 genes (Luc-HCT116^CCR6^). ZsGreen1^+^ Luc-HCT116 cells or ZsGreen1^+^ CCR6^+^ Luc-HCT116 cells were further purified by fluorescence activated cell sorting and expanded in the culture media.

### Human Tumor Metastasis PCR Array

HCT116^Ctr^ or HCT116^CCR6^ cells were lysed directly in TRIzol reagent, and total RNA was extracted according to the manufacturer’s instructions (Invitrogen, Carlsbad, CA). Subsequently, The Super Script III synthesis system (Invitrogen) was employed for cDNA synthesis. Real-time PCR was performed on each sample using the Human Tumor Metastasis RT^2^ Profiler PCR Array (Super Array Bioscience, USA) on an ABI 7900HT fast real-time PCR system (Applied Biosystems, USA). Human β-Actin was employed for normalization by the ΔΔCt method.

### Experimental *in vivo* Liver Metastasis Model

Liver metastatic capacity was determined by injecting 1×10^6^ cells per mouse into the spleen of BALB/c nude mice. Briefly, BALB/c nude mice were anesthetized by i.p. injection of Pelltobarbitalum Natricum, and 1×10^6^ HCT116^Ctr^ or HCT116^CCR6^ tumor cells in 25 µL were injected into the exteriorized spleen with an insulin syringe (BD company) after abdominal incision. Five minutes after cell injection, spleen blood vessels were ligated, and the spleen was removed. Finally, the abdominal wound was closed with staples. After 5 weeks, mice were sacrificed and the livers were then removed and photographed.

### Experimental *in vivo* Lung Metastasis Model

Seven 7-week-old male BALB/c nude mice in each group were injected with Luc-HCT116^Ctr^ or Luc-HCT116^CCR6^ cells. Briefly, 5×10^6^ cells suspended in 200 µl PBS were injected into the tail vein of each BALB/c nude mouse. After 6 weeks, animals were sacrificed and examined macroscopically and microscopically for the presence of metastases. For whole-body small animal imaging, mice were given 150 µg/g of D-luciferin substrate in sterile PBS by intraperitoneal injection and then anesthetized with isoflurane. Bioluminescence images were captured with a charge-coupled device (Xenogen IVIS 200 System, Xenogen Inc., Hopkinton, MA, USA) within 15 min after substrate injection.

### Syngeneic Graft CRC Model and anti-CCR6 Therapy

CMT93 cells in 100 µl PBS were injected subcutaneously (s.c.) into 6-week-old male CCR6^−/−^ mice (1×10^6^ cells/mouse), or CT26 cells in 100 µl PBS were injected s.c. into 7-week-old male BALB/c mice (1×10^6^ cells/mouse). Ten days after tumor cell inoculation, when the tumor cells formed solid tumor tissues, grafted CCR6^−/−^ mice or BALB/c mice were randomly divided into two groups for treatment with IgG control or anti-CCR6. Twenty micrograms of IgG_2A_ (R&D System, Clone #54447) dissolved in 100 µl PBS or 20 µg CCR6 (R&D System, Clone #140706) dissolved in 100 µl PBS was separately injected into the tumor sites every other day for 18 days. After 28 days, the mice were sacrificed, and the xenografts were removed, photographed and weighed. For Western blot analysis, polyclonal anti-mouse CCR6 (#ab78429, Abcam) was used as the primary antibody.

### Statistical Analysis

Statistical analysis was performed with GraphPad Prism 5.01 software. Statistical tests for data analysis included the log-rank test, Fisher’s exact test, the Chi-square test, the Wilcoxon test and the U-Mann Whitney test. Multivariate statistical analysis was performed using a Cox regression model. The quantitative data were presented as the mean values ± standard deviations (SD). Differences were considered to be statistically significant at values of *p<0.05, **p<0.01, and ***p<0.001.

## Results

### Upregulation of CCR6 is Correlated with Tumor Progression

**Figure 1:**
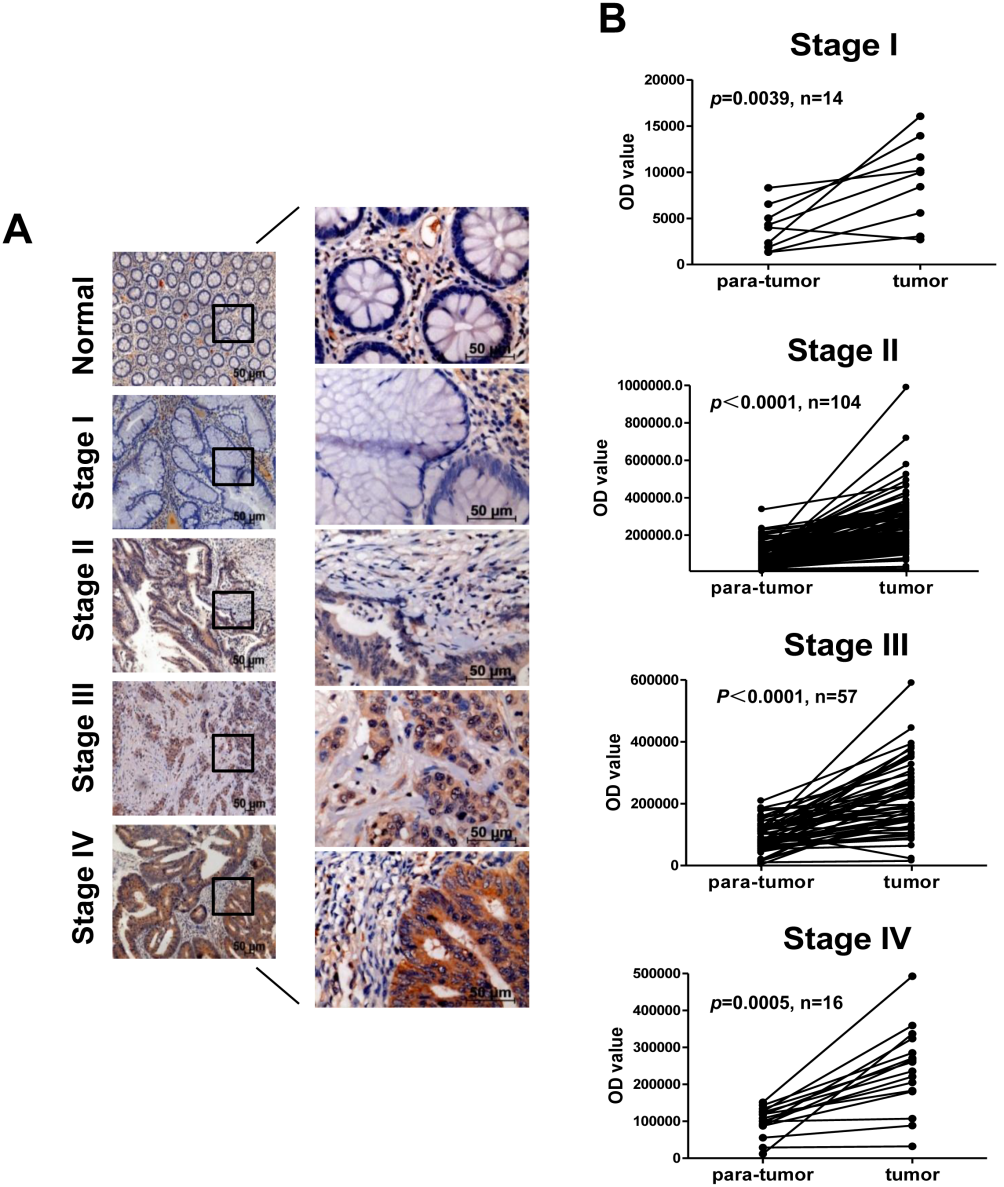
CCR6 is Upregulated in Primary Human CRC Samples. (A) Immunohistochemical staining of CCR6 in primary CRC derived from 191 CRC patients with clinical stage I–IV. (B) Digital image analysis was performed to count staining intensity of CCR6 area fraction (CCR6-AF) values of paired para-tumor/tumor samples in each clinical stage, Wilcoxon test.

To investigate the clinical relevance of upregulated CCR6 in CRC, we collected 191 paraffin-embedded primary CRC tissue samples (**Table S1**), and CCR6 expression levels were examined in all of these samples using immunohistochemistry. The correlation of CCR6 expression with clinicopathological features and the survival of CRC patients were summarized (**Table S2**). Statistical analyses revealed that CCR6 expression was significantly correlated with the clinical stage (*p* = 0.0117), N classification (*p* = 0.0309), M classification (*p* = 0.0334) and vital status (*p* = 0.0019) in patients with CRC (**Table S2**). Moreover, we found that CCR6 expression was markedly upregulated in all assayed clinical CRC samples but was only detectable at low levels in paratumor tissue (**Figure 1A**), and univariate analysis revealed a strong association between CCR6 staining intensity and tumor stages (n = 14, *p* = 0.0039; n = 104, *p*<0.0001; n = 57, *p*<0.0001; n = 16, *p* = 0.0005) (**Figure 1B**). These data suggest that CCR6 upregulation is strongly associated with the clinical progression of human CRC.

### Upregulated CCR6 Indicates Poor Prognosis for CRC Patients

**Figure 2:**
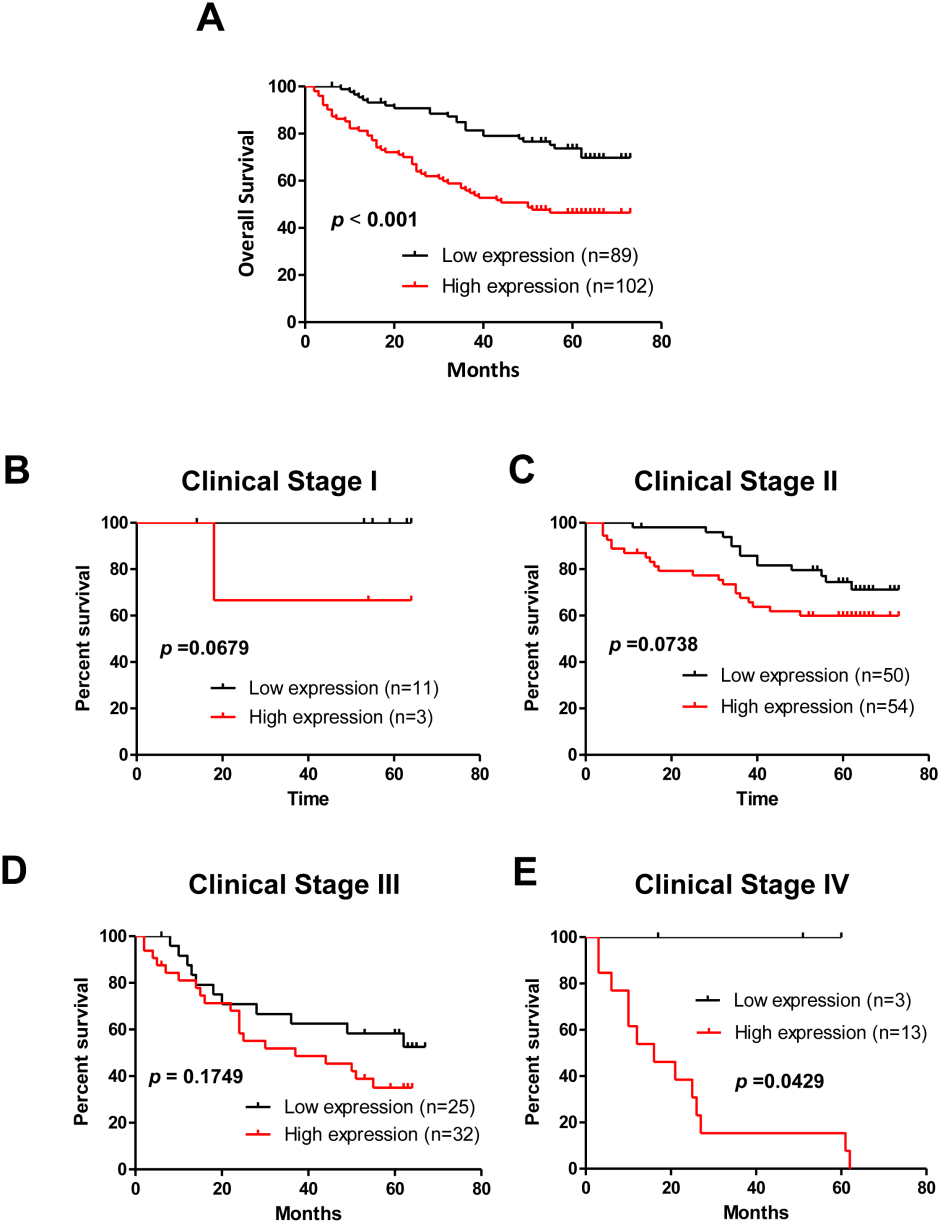
Survival Analysis of CRC Patients with Low versus High Expression of CCR6. (A) Kaplan-Meier curves of CRC patients with low versus high expression of CCR6 (n = 191, *p*<0.001, log-rank test). (B) Kaplan-Meier curves of CRC patients with low versus high expression of CCR6 in clinical stage I (n = 14, *p* = 0.0679, log-rank test). (C) Kaplan-Meier curves of CRC patients with low versus high expression of CCR6 in clinical stage II (n = 104, *p* = 0.0738, log-rank test). (D) Kaplan-Meier curves of CRC patients with low versus high expression of CCR6 in clinical stage III (n = 57, *p* = 0.1749, log-rank test). (E) Kaplan-Meier curves of CRC patients with low versus high expression of CCR6 in clinical stage IV (n = 16, *p* = 0.0429, log-rank test).

Importantly, we demonstrated that the CCR6 expression was inversely correlated with the survival time (*p*<0.001) (**Figure 2A**). Furthermore, CCR6 expression was correlated with overall survival for subgroup of patients at different clinical stage, significantly at stage IV (*p*<0.05) (**Figure 2B–E**). In addition, univariate and multivariate analyses revealed that M classification and CCR6 expression were each recognized as independent prognostic factors in CRC (**Table S3**). Together, these data suggest that CCR6 has potential clinical value as a predictive biomarker for disease outcome in CRC patients.

### Knockdown of CCR6 Inhibits the Migration of CRC Cells *in vitro*

**Figure 3:**
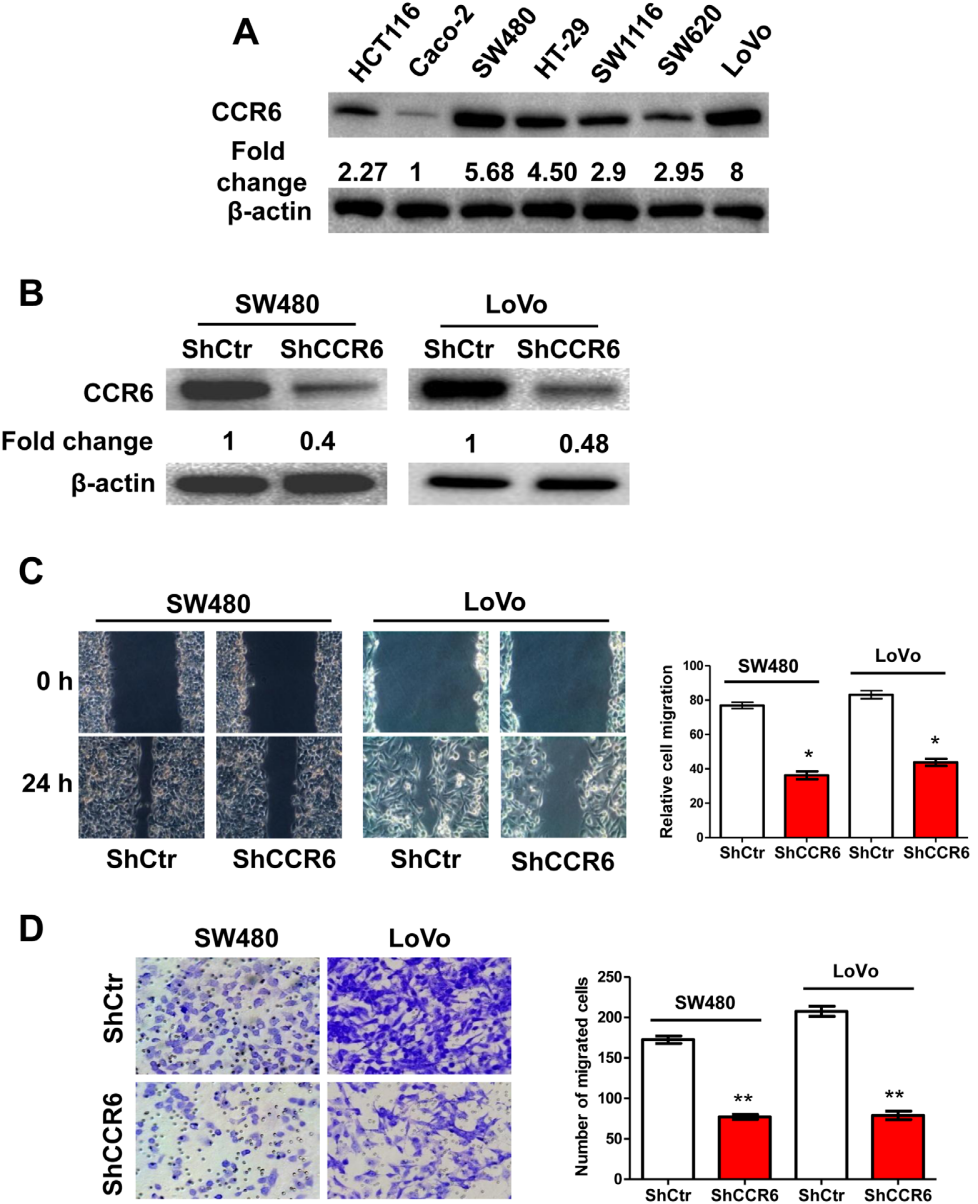
Knockdown of CCR6 by shRNA Inhibits CRC Cell Migration *in vitro*. (A) Western blotting analysis of CCR6 levels in 7 cultured CRC cell lines. Values were expressed as fold changes relative to Caco-2, and normalized to β-actin. (B) Western blotting analysis of knockdown of CCR6 in SW480 and LoVo cells, β-actin served as a loading control. Values were expressed as fold changes relative to controls (ShCtr), and normalized to β-actin. (C) Wound-healing assays for motility of CCR6-silenced SW480 and LoVo cells and control cells. Representative pictures of one field at the beginning (t = 0 hr) (upper panel) and at the end of the recording (t = 24 hr) (lower panel) in each condition are shown. The relative cell migration in ShCtr and shCCR6 groups are shown in the right panel. (D) Representative images of transwell migrated cells in CCR6-silenced SW480 and LoVo cells (lower panel) or control cells (upper panel) cells. The numbers of migrated cells in ShCtr and shCCR6 groups are shown in the right panel. Values represent mean from triplicate wells, ± S.D. *p<0.05, **p<0.01, Wilcoxon test. Data are representative of at least three independent experiments.

The observed CCR6 overexpression had a strong association with CRC progression, which prompted us to investigate the impact of CCR6 on CRC cell migration capability. Two CRC cell lines, SW480 and LoVo, which expressed the highest CCR6 expression of colorectal cell lines we analyzed, were used to create sublines with CCR6 stably knocked down by shRNA (**Figure 3A, B**). Strikingly, the results showed that the knockdown of CCR6 caused a significant suppression of cell migration in both SW480 and LoVo cell lines in a wound-healing assay (*p*<0.05, **Figure 3C**). We also used another classical transwell assay to assess the contribution of CCR6 on the cell migration. We demonstrated that the ablation of CCR6 markedly reduced the migration of both SW480 and LoVo cell lines (*p*<0.01, **Figure 3D**). Taken together, our data indicated that the specific knockdown of CCR6 in CRC cell lines could significantly reduce cell migration *in vitro.*

### CCR6 Promotes the Aggressiveness of CRC Cells *in vitro*

**Figure 4:**
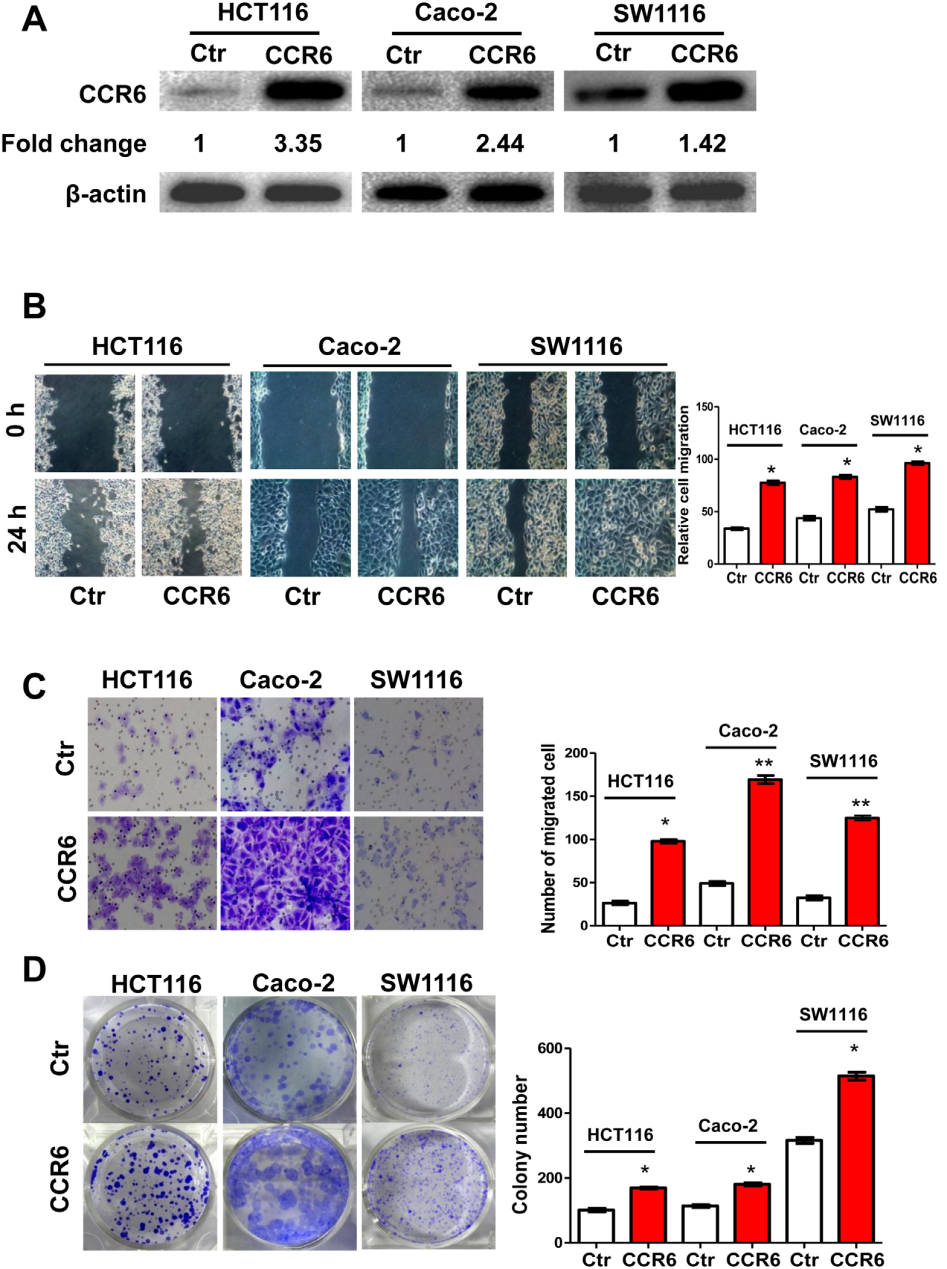
Enhanced Proliferation and Migration of CRC cells with Overexpressed CCR6. (A) Western blotting analysis of ectopic expression of CCR6 in HCT116^Ctr^ and HCT116^CCR6^ cells or Caco-2^Ctr^ and Caco-2^CCR6^ or SW1116^Ctr^ and SW1116^CCR6^ cells. β-actin served as a loading control. Values were expressed as fold changes relative to controls (Ctr), and normalized to β-actin. (B) Wound-healing assay for motility of HCT116^Ctr^ and HCT116^CCR6^ or Caco-2^Ctr^ and Caco-2^CCR6^ or SW1116^Ctr^ and SW1116^CCR6^ cells. Representative pictures of one field at the beginning (t = 0) (upper panel) and at the end of the recording (t = 24 h) (lower panel) in each condition are shown. The relative cell migration in CCR6 and control groups are shown in the right panel. (C) Representative images of transwell migrated cells in stably transfected HCT116^Ctr^, Caco-2^Ctr^, SW1116^Ctr^ (upper panel) or HCT116^CCR6^, Caco-2^CCR6^, SW1116^CCR6^ (lower panel) cells. Average number of migrated cells of HCT116^Ctr^ and HCT116^CCR6^ or Caco-2^Ctr^ and Caco-2^CCR6^ or SW1116^Ctr^ and SW1116^CCR6^ cells are shown in the right panel. (D) Representative image of colony formation in HCT116^Ctr^, Caco-2^Ctr^, SW1116^Ctr^ (upper panel) or HCT116^CCR6^, Caco-2^CCR6^, SW1116^CCR6^ cells (lower panel). Values represent mean from triplicate wells, ± S.D. *p<0.05, **p<0.01, Wilcoxon test. Data are representative of at least three independent experiments.

To investigate whether the ectopic overexpression of CCR6 could enhance the aggressiveness of CRC cells, HCT116, Caco-2 and SW1116 cell lines stably expressing ectopic CCR6 were established (**Figure 4A**). Strikingly, both wound healing and migration chamber assays revealed that CCR6 overexpression pronouncedly enhanced cell migration compared to control groups (*p*<0.05 or *p*<0.01) (**Figure 4B, C**). In addition, during the process of establishing CCR6 stable cell lines, we noticed that there was more colony formation in CCR6-transfected cells than control transfected cells. We therefore performed colony formation assays using the stably transfected HCT116^CCR6^, HCT116^Ctr^, Caco-2^Ctr^, Caco-2^CCR6^, SW1116^Ctr^ and SW1116^CCR6^ cells. Again, we noted that compared to control (Ctr) cells, the size and number of colonies formed in CCR6 overexpressing HCT116, Caco-2 and SW1116 cells were significantly increased (*p*<0.05) (**Figure 4D**). Taken together, our data provide evidence that the elevated expression of CCR6 plays a critical role in the aggressive phenotype of CRC cells *in vitro*.

### Overexpression of CCR6 Facilitates Metastasis of CRC Cells *in vivo*

**Figure 5:**
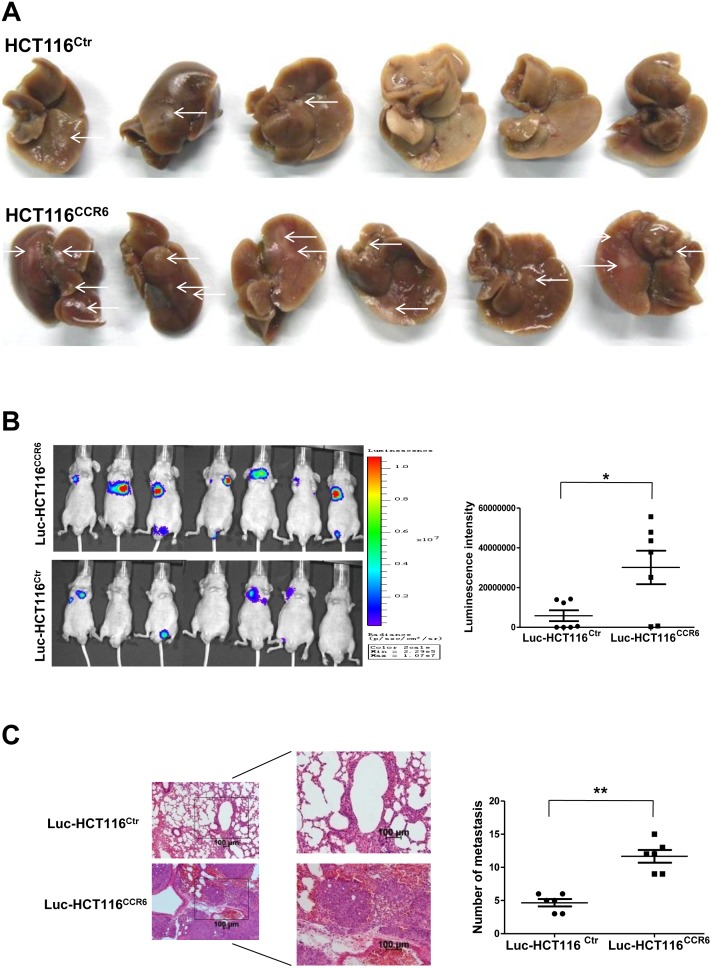
Increased Metastasis of CRC Cells with Overexpressed CCR6 *in vivo*. (A) Number of metastatic nodules (indicated by white arrows) formed in the liver of BALB/c nude mice 5 weeks after spleen injection of HCT116^Ctr^ (upper panel) or HCT116^CCR6^ (lower panel) cells (six mice per group). (B) *In vivo* metastasis assays of Luc-HCT116^Ctr^ and Luc-HCT116^CCR6^ cells by tail vein injection. The whole body metastasis burden of xenografted animals was monitored at 6 weeks after CRC cell injection using the IVIS Imaging System. Statistical analysis of luciferase intensity from mice injected with Luc-HCT116^Ctr^ or Luc-HCT116^CCR6^ cells was shown in the right panel. (C) Representative images of H&E staining of lungs prepared from mice injected with Luc-HCT116^Ctr^ or Luc-HCT116^CCR6^ cells at ×5 (left panel) and ×10 (right panel) magnification. Statistical analysis of the number or mitosis by mm^2^ in each metastatic nodule in the five lung H&E staining from mice injected with Luc-HCT116^Ctr^ or Luc-HCT116^CCR6^ cells was shown in the right panel. *p<0.05, **p<0.01, U-mann whitney test.

The liver is the most common site of colorectal cancer metastasis. To determine whether CCR6 specifically plays an important role in the liver metastasis of CRC, we established an experimental *in vivo* liver metastasis model by injecting human tumor cells into the spleens of BALB/c nude mice and followed their ability to invade via the portal vein into the liver to form metastases. To define the relationship between CCR6 and CRC liver metastasis *in vivo*, six 7-week-old male BALB/c nude mice in each group were injected with HCT116^Ctr^ or HCT116^CCR6^ cells into the spleen before splenectomy. After 5 weeks, the mice were killed, and the metastatic tumor nodules that formed in the liver were examined. Strikingly, metastatic tumor nodules were more frequently found in the livers of the HCT116^CCR6^ group than the HCT116^Ctr^ group (**Figure 5A**, **indicated by white arrows**). These results suggest that CCR6 overexpression in cancer cells can enhance liver metastases of CRC. Additionally, we used the whole-body small animal fluorescence imaging system (IVIS) to monitor the migration of tumor cells *in vivo*. First, we constructed the stably expressing luciferase cell line, luciferase-HCT116 by transfecting HCT116 cells with the luciferase lentivirus, and selected these cells with G418. Thereafter, we constructed two cell lines with or without CCR6 overexpression by transfecting luciferase-HCT116 cells with control-lentivirus or CCR6-lentivirus to create Luc-HCT116^Ctr^ and Luc-HCT116^CCR6^ cells. To define the relationship between CCR6 and CRC cell lung metastasis *in vivo*, Luc-HCT116^Ctr^ or Luc-HCT116^CCR6^ cells were transplanted into BALB/c nude mice via tail-vein injection. Six weeks after injection, the group of Luc-HCT116^CCR6^ had increased metastasis compared to the group of Luc-HCT116^Ctr^ (*p*<0.05) (**Figure 5B**). H&E staining of lungs confirmed that the number and size of micrometastases were significantly greater in Luc-HCT116^CCR6^ mice than in Luc-HCT116^Ctr^ mice (*p*<0.01) (**Figure 5C**). Collectively, our data suggest that CCR6 is a critical factor for promoting the metastasis of CRC cells *in vivo*.

### CCR6 Activates the Akt Signaling Pathway and Affects Metastasis-related Genes

**Figure 6:**
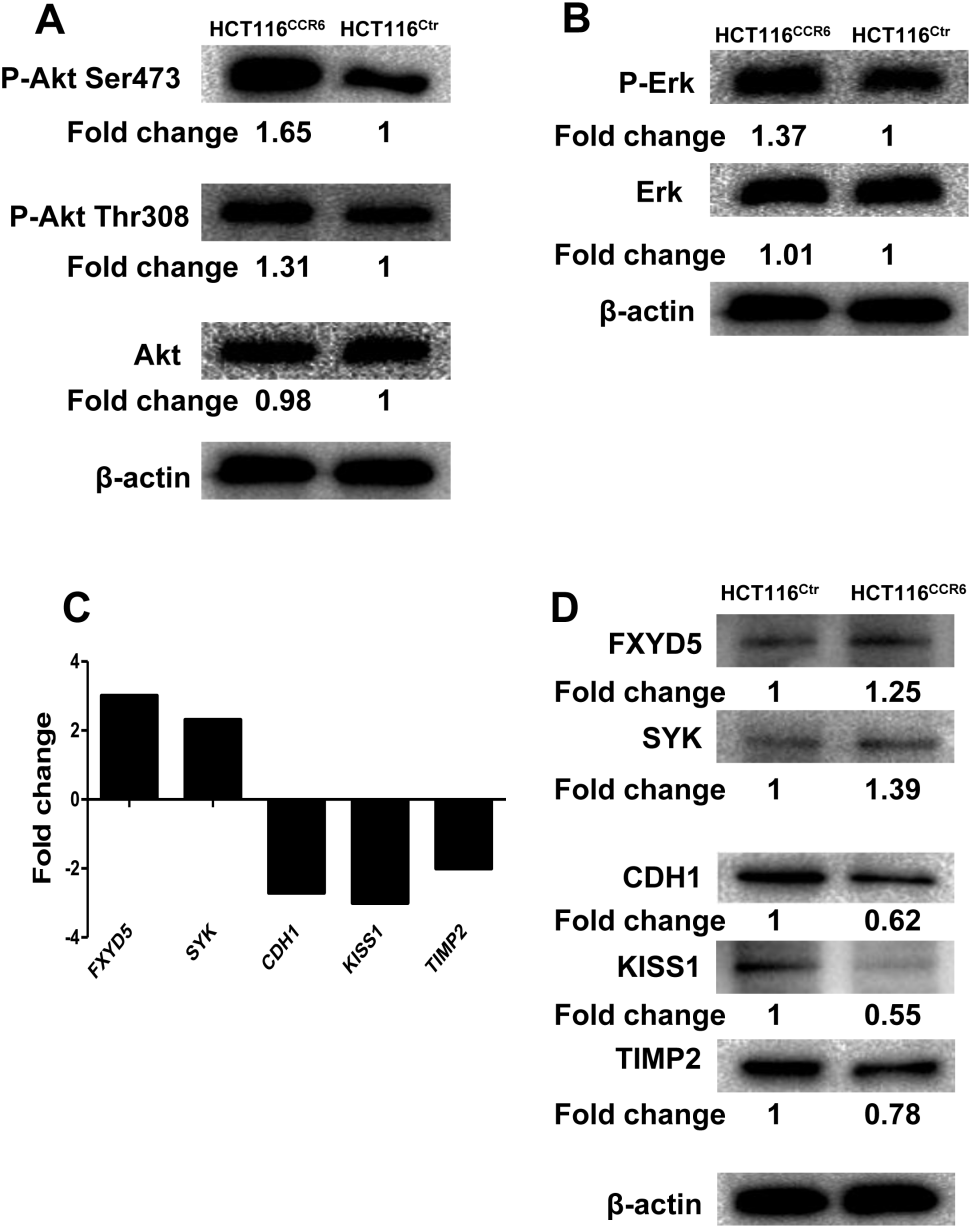
Signaling Pathway Involved in the Aggressiveness of HCT116^CCR6^. (A, B) Western blotting analysis of Erk1/2 or phospho-Erk1/2 and Akt, phosphorylated Akt (Ser473) or phosphorylated Akt (Ser308) in HCT116^CCR6^ and HCT116^Ctr^ cells. Values were expressed as fold changes relative to HCT116^Ctr^, and normalized to β-actin. (C) Upregulated (FXYD5 and SYK) or down-regulated genes (CDH1, KISS1 and TIMP2) in HCT116^CCR6^ cells screening with a human tumor metastasis RT^2^ profiler PCR Array. (D) Western blotting analysis of changed FXYD5, SYK, CDH1, KISS1 and TIMP2 genes in HCT116^CCR6^ and HCT116^Ctr^ cells. Values were expressed as fold changes relative to HCT116^Ctr^, and normalized to β-actin.

A previous study has shown that the activation of CCR6 by CCL20 resulted in the activation of extracellular signal-regulated kinase (ERK) and Akt signaling pathways in SW480 cells, and the activation of ERK and Akt after CCL20 stimulation resulted in an increase of cell proliferation and migration [23]. Herein, we examined whether these pathways were involved in the aggressiveness of HCT116^CCR6^ cells. To this end, we performed western blotting using a specific antibody against phosphorylated Akt (Ser473) in HCT116^CCR6^ cells. We demonstrated that the level of phospho-Ser473 Akt was increased in HCT116^CCR6^ cells compared with HCT116^Ctr^ cells (**Figure 6A**). The overexpression of CCR6 resulted in only a weak increase of phospho-Thr308 Akt and ERK phosphorylation (**Figure 6A, B**), indicating that the overexpression of CCR6 primarily activated the phosphorylation of Akt at Ser473 in HCT116 cells. Thus, these data suggest that the upregulation of CCR6 contributes to the aggressiveness of HCT116^CCR6^ cells, likely by activating the Akt signaling pathway. To further explore the molecular mechanisms of upregulated CCR6 in CRC cell migration and/or metastasis, the mRNA expression profiles of HCT116^CCR6^ and HCT116^Ctr^ cells were analyzed using a human tumor metastasis RT^2^ profiler PCR array containing 84 cell metastasis-related genes. Our data identified 2 upregulated metastasis genes (FXYD5 and SYK) and 3 downregulated metastasis suppressor genes (CDH1, KISS1 and TIMP2) that showed more than a two-fold change in mRNA levels in HCT116^CCR6^ cells compared to HCT116^Ctr^ cells (**Table S4 and** **Figure 6C**). Subsequently, these metastasis-related genes were selected and further validated by western blotting. Consistent with mRNA levels, overexpression of CCR6 led to upregulated FXYD5, SYK, and downregulated CDH1, KISS1, TIMP2 protein levels in HCT116 cells (**Figure 6D**). Thus, these data imply that overexpression of CCR6 in CRC likely upregulated metastasis genes and downregulated metastasis suppressor genes to enhance the aggressiveness of CRC cells.

### Targeting Tumor-expressing CCR6 Inhibits CRC Progression

**Figure 7:**
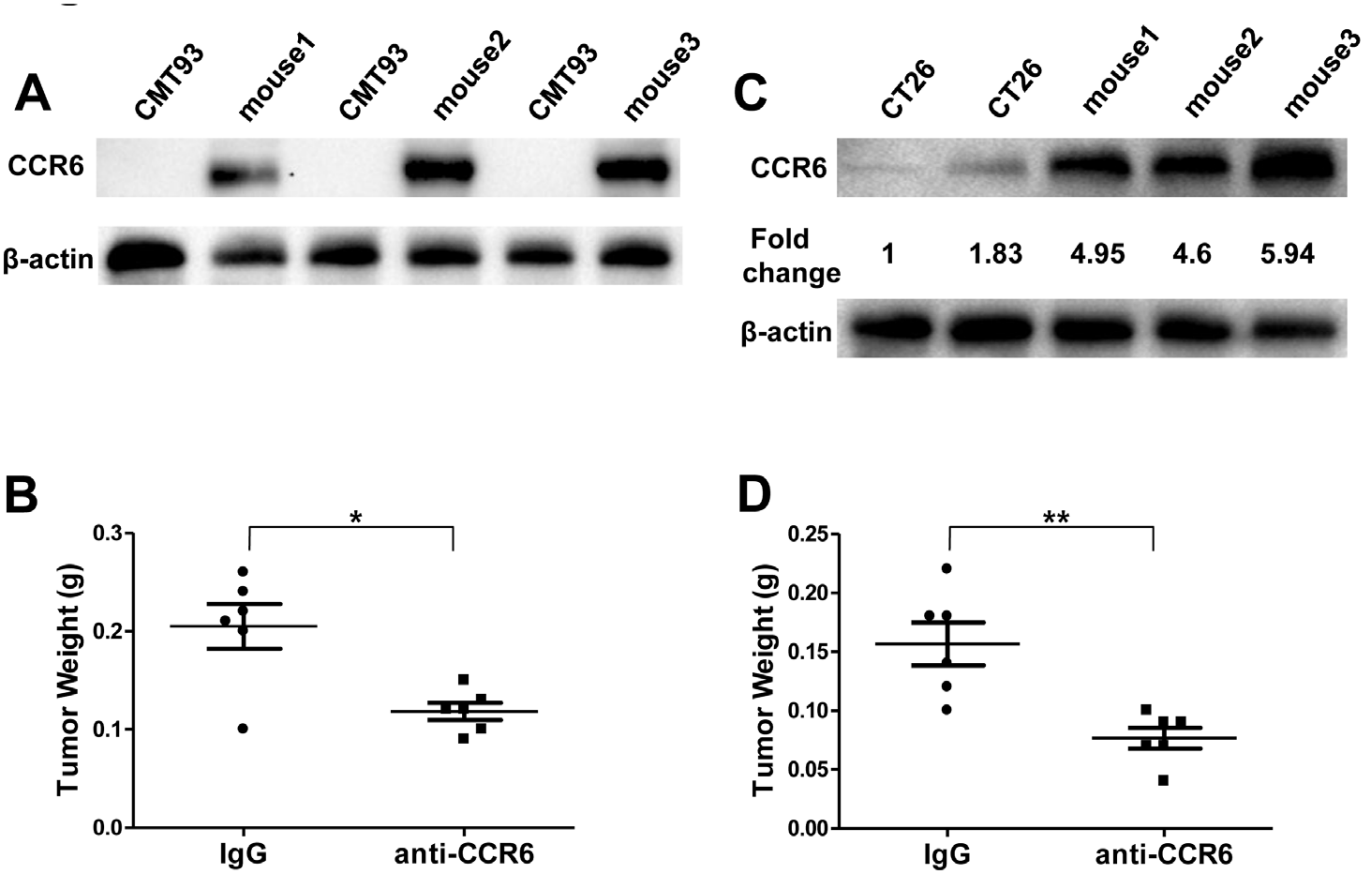
Inhibition of Mouse CRC Progression by Targeting Tumor-expressing CCR6. (A) Western blotting analysis of CCR6 in murine CMT93 colorectal tumor cell line and CRC tissue derived from CCR6^−/−^ mice grafted with CMT93 at day10. (B) Statistical analysis of tumor weight in each group treated with IgG or anti-CCR6. (C) Western blotting of CCR6 in murine CT26 colorectal tumor cell line and CRC tissue derived from Balb/c mice grafted with CT26 at day 10. (D) Statistical analysis of tumor weight in each group treated with IgG or anti-CCR6. *p<0.05, **p<0.01, U-mann whitney test.

We next determined the *in vivo* functionality of CCR6 in subcutaneous xenograft mouse models. We injected mouse CMT93 colorectal cancer cells s.c. into CCR6^−/−^ mice to exclude the effect of CCR6 expressed by other immune cells in hosts. We found that CMT93 cells strongly expressed CCR6 *in vivo* after being grafted into CCR6^−/−^ mice for 10 days (**Figure 7A**), suggesting that CCR6 is potentially involved in CRC progression. Ten days after tumor cell inoculation, the grafted CCR6^−/−^ mice were randomly divided into two groups for treatment, IgG control or anti-CCR6. We observed that the anti-CCR6 antibody significantly inhibited the CRC growth compared to IgG (*p*<0.05) (**Figure 7B**). We next injected mouse CT26 colorectal cancer cells s.c. into Balb/c mice. Similar to CMT 93, murine CT26 cells displayed low CCR6 expression before grafting to hosts, however CCR6 was strongly expressed in the tumor tissues 10 days post injection (**Figure 7C**). We again found that anti-CCR6 antibody significantly inhibited the CT26 growth compared to IgG (*p*<0.01) (**Figure 7D**). Thus, our data suggest that blocking CCR6 will be an effective therapeutic strategy for the treatment of CRC.

## Discussion

It is commonly believed that during CRC metastasis, cancer cells must overcome a number of hurdles, including invasion into adjacent tissues, intravasation into blood or lymphatic vessels, survival in the circulation, extravasation from vessels at distant organs, colonization and, finally formation of clinically detectable metastases. Each of these events involves a number of signaling pathways [24]. Recently, it has been proposed that chemokine receptors play a critical role in determining the metastatic destination of tumor cells [15], [16], [25], [26]. The chemokine receptor CCR6 is of particular interest in CRC metastasizing to the liver [20]. Its sole ligand CCL20, which was originally identified in the liver and called liver-and activation-related chemokine, is the only chemokine known to interact with CCR6 and is primarily expressed in the liver, the most frequent metastasis site of CRC [20].

Herein we demonstrate that CCR6 plays a critical role in CRC cell aggressiveness both *in vitro* and *in vivo* settings, indicating that CCR6 on tumor cells is functional. Our study is consistent with previous research which revealed that high expression of CCR6 in primary CRC was strongly associated with synchronous liver metastases [20]. Moreover, our results represented the first large-scale analysis of CCR6 expression that was closely associated with a reduced survival time in CRC patients. Thus, upregulated CCR6 may be important in the acquisition of an aggressive phenotype for CRC. The multivariate analysis showed that CCR6 is an independent risk factor for liver metastasis. However, large-cohort studies in a multicenter setting will be necessary to validate these findings and examine potential mechanisms for decreased survival time. The use of antibody blocking the CCR6 receptor has shown promise for future therapeutic strategies that may allow for controlling tumor metastasis facilitated by the CCR6 receptor.

It was previously showed that both pancreatic cancer cells and tumor-associated macrophages are *in vivo* sources of CCL20 mRNA [27], and CRC cell lines expressed transcripts for both CCL20 and its receptor CCR6 [23]. Therefore, in addition to its potential role in the recruitment of tumor infiltrating lymphocytes or tumor-associated immature DCs, CCL20 may also contribute to tumor cell growth and migration via autocrine and paracrine mechanisms [6], [10]. We did not address whether CCR6 promotes CRC cell aggressiveness through an autocrine or paracrine manner or both. However, according to homing chemokine theory, CRC cells expressing CCR6 likely seed to distant sites where high levels of CCL20 are found. Thus, we propose that once tumor cells express CCR6, their migration and metastasis behavior could be greatly enhanced by stimulating with CCL20 produced by macrophages or other immune cells in the tumor microenvironment. Once the tumor cells have entered the blood or lymphatic vessels, the constitutive expression of CCL20 by the liver or other sites attracts a second wave of CCR6-expressing CRC cell migration. The liver may then selectively attract cells to attach and form micrometastases, perhaps by binding to putative integrin-like adhesion molecules that are possibly either induced or activated on cancer cells via CCL20/CCR6 interaction.

The primary consequence of PI3K activation is to catalyze the conversion of membrane-bound PIP2 to PIP3 [28]. As a second messenger, PIP3 works as a ligand to recruit PH domain-containing proteins (most notably the serine-threonine protein kinase Akt1 and phosphoinositide-dependent kinase 1 (PDK1)) to the inner surface of cell membrane. Once positioned at the cell membrane, Akt1 is activated by PDK1 through the phosphorylation of threonine 308, which is in the activation loop of Akt [28]. The full repertoire of Akt functions is then accessible when it undergoes phosphorylation at serine 473. Once activated, Akt is poised to serve as a central node for regulating a variety of cellular functions, including but not limited to proliferation, cell survival, metabolism, and angiogenesis [28], [29]. Recent studies have shown that the PI3K/Akt signaling pathway is aberrantly activated in many cancer types, including CRC and that the activation of PI3K signaling promotes cancer formation through a variety of mechanisms, including the induction of cell proliferation, migration and cancer cell survival [30]–[32]. In this study, we found that CCR6 enhanced the aggressiveness of CRC cells partly through PI3K activation.

To further elucidate the downstream signaling pathway involving upregulated CCR6 in CRC aggressiveness, we compared mRNA expression profiles between HCT116^CCR6^ and HCT116^Ctr^ cells using a human tumor metastasis real-time PCR array containing 84 genes known to be involved in metastasis. Genes selected for this array encode several classes of protein factors including cell adhesion, ECM components, cell cycle, cell growth and proliferation, apoptosis, transcription factors and regulators, and other genes related to tumor metastasis. These genes can mimic all aspects of the metastatic development. Among the 84 genes, we identified 5 genes with different expression (upregulated: FXYD5 and SYK; downregulated: CDH1, KISS1 and TIMP2). FXYD5 overexpression in pancreatic ductal adenocarcinoma reflects tumor aggressiveness and promotes metastasis [33]. Syk has been shown to mediate chemomigration in nasopharyngeal carcinoma cells [34]. The down-regulation of E-cadherin (CDH1) is considered as a critical event for the invasion and metastasis of colorectal carcinoma, and the loss of E-cadherin-mediated cell adhesion is one rate-limiting step in the progression from adenoma to carcinoma [35]. KISS1 is a gene that suppresses the metastasis of tumor cells without affecting tumorigenicity [36]. The proteins encoded by the TIMP2 gene family are natural inhibitors of the matrix metalloproteinases, a group of peptidases involved in the degradation of the extracellular matrix [37]. Our data suggest that overexpressed CCR6 likely upregulated metastasis genes and downregulated metastasis suppressor genes to enhance the aggressiveness of HCT116^CCR6^ cells.

To date, there are no small molecule programs in clinical development for anti-CCR6 therapy for CRC treatment. In this study, we demonstrated that targeting the CCR6 in the tumor cell or the tumor microenvironment inhibited CRC progression in mice. Thus, our findings highlight CCR6 as a promising therapeutic target for CRC. In addition, we have shown that upregulated CCR6 enhanced CRC cell proliferation, migration and *in vivo* tumor metastasis, likely by altering the expression of tumor metastasis-related genes. However, the identification of tumor-metastasis-related genes regulated by the CCR6/CCL20 axis will be clinically important for designing targeted therapies for the treatment of CRC in the future.

## Supporting Information

Table S1 **Clinicopathologic characteristics of studied patients and expression of CCR6 in CRC.**
(DOCX)

Table S2 **Correlation between clinicopathological features and expression of CCR6.**
(DOCX)

Table S3 **Univariate and multivariate analysis of different prognostic parameters in patients with CRC by Cox-regression analysis.**
(DOCX)

Table S4 **List of genes differentially expressed in HCT116^CCR6^ and HCT116^Ctr^ cell lines using a human tumor metastasis real-time PCR array.**
(DOCX)
