# Supplementary material for: CCR6 Is a Prognostic Marker for Overall Survival in Patients with Colorectal Cancer, and Its Overexpression Enhances Metastasis In Vivo
Source: PLoS One. 2014 Jun 30;9(6):e101137. doi: 10.1371/journal.pone.0101137 (PMC4076197; doi:10.1371/journal.pone.0101137)
Supplement: Table S1 — Clinicopathologic characteristics of studied patients and expression of CCR6 in CRC. (DOCX) [file pone.0101137.s001.docx]

**Table S1**

| **Factor** | **No. of Patients (N=191)** | **%** |
| --- | --- | --- |
| **Age** |  |  |
| <70 years | 103 | 53.9 |
| ≥70 years | 88 | 46.1 |
| **Sex** |  |  |
| Male | 98 | 51.3 |
| Female | 93 | 48.7 |
| **Location** |  |  |
| Colon | 191 | 100 |
| **Tissue samples** |  |  |
| Primary | 191 | 100 |
| UICC/AJCC stage |  |  |
| pI | 14 | 7.3 |
| pII | 104 | 54.5 |
| pIII | 57 | 29.8 |
| pIV | 16 | 8.4 |
| **Pathologic grade** |  |  |
| Well | 31 | 16.2 |
| Moderate | 106 | 55.5 |
| Poor | 54 | 28.3 |
| **T classification** |  |  |
| T1 | 3 | 1.6 |
| T2 | 16 | 8.4 |
| T3 | 142 | 74.3 |
| T4 | 30 | 15.7 |
| **N classification** |  |  |
| N0 | 118 | 61.8 |
| N1 | 53 | 27.7 |
| N2 | 20 | 10.5 |
| **M classification** |  |  |
| No | 175 | 91.6 |
| Yes | 16 | 8.4 |
| **Vital status** |  |  |
| Alive | 112 | 58.6 |
| Dead | 79 | 41.4 |
| **Expression of CCR6** |  |  |
| Low expression | 89 | 46.6 |
| High expression | 102 | 53.4 |
