# Supplementary material for: CCR6 Is a Prognostic Marker for Overall Survival in Patients with Colorectal Cancer, and Its Overexpression Enhances Metastasis In Vivo
Source: PLoS One. 2014 Jun 30;9(6):e101137. doi: 10.1371/journal.pone.0101137 (PMC4076197; doi:10.1371/journal.pone.0101137)
Supplement: Table S2 — Correlation between clinicopathological features and expression of CCR6. (DOCX) [file pone.0101137.s002.docx]

**Table S2**

| **Patient characteristics** | **Expression of CCR6** | | ***p* value** |
| --- | --- | --- | --- |
|  | **Low** | **High** |  |
| **Age** |  |  |  |
| <70 years | 48 | 55 | NS |
| ≥70 years | 41 | 47 |  |
| **Sex** |  |  |  |
| Male | 48 | 50 | NS |
| Female | 41 | 52 |  |
| **UICC/AJCC stage** |  |  |  |
| pI | 11 | 3 | 0.0117 |
| pII | 50 | 54 |  |
| pIII | 25 | 32 |  |
| pIV | 3 | 13 |  |
| **Pathologic grade** |  |  |  |
| Well | 16 | 15 | NS |
| Moderate | 55 | 51 |  |
| Poor | 18 | 36 |  |
| **T classification** |  |  |  |
| T1 | 3 | 0 | NS |
| T2 | 10 | 6 |  |
| T3 | 62 | 80 |  |
| T4 | 14 | 16 |  |
| **N classification** |  |  |  |
| N0 | 61 | 57 | 0.0309 |
| N1 | 24 | 29 |  |
| N2 | 4 | 16 |  |
| **M classification** |  |  |  |
| No | 86 | 89 | 0.0334 |
| Yes | 3 | 13 |  |
| **Vital status** |  |  |  |
| Alive | 63 | 49 | 0.0019 |
| Dead | 26 | 53 |  |
