# Supplementary material for: CCR6 Is a Prognostic Marker for Overall Survival in Patients with Colorectal Cancer, and Its Overexpression Enhances Metastasis In Vivo
Source: PLoS One. 2014 Jun 30;9(6):e101137. doi: 10.1371/journal.pone.0101137 (PMC4076197; doi:10.1371/journal.pone.0101137)
Supplement: Table S3 — Univariate and multivariate analysis of different prognostic parameters in patients with CRC by Cox-regression analysis. (DOCX) [file pone.0101137.s003.docx]

**Table S3**

|  | **Univariate analysis** | | | **Multivariate analysis** | | |
| --- | --- | --- | --- | --- | --- | --- |
|  | **No. patients** | ***p* value** | **Regression coefficient (SE)** | ***p* value** | **Relative risk** | **95% confidence interval** |
| **M classification** |  |  |  |  |  |  |
| M_0_ |  | < .001 | 0.307 | < .001 | 3.185 | 1.733-5.852 |
| M_1_ |  |  |  |  |  |  |
| **Expression of CCR6** |  |  |  |  |  |  |
| Low |  | < .001 | 0.240 | 0.002 | 2.141 | 1.331-3.444 |
| High |  |  |  |  |  |  |
