# Supplementary material for: CCR6 Is a Prognostic Marker for Overall Survival in Patients with Colorectal Cancer, and Its Overexpression Enhances Metastasis In Vivo
Source: PLoS One. 2014 Jun 30;9(6):e101137. doi: 10.1371/journal.pone.0101137 (PMC4076197; doi:10.1371/journal.pone.0101137)
Supplement: Table S4 — List of genes differentially expressed in HCT116CCR6 and HCT116Ctr cell lines using a human tumor metastasis real-time PCR array. (DOCX) [file pone.0101137.s004.docx]

**Table S4**

| **Gene** | **Fold change** | **Location** | **Function** |
| --- | --- | --- | --- |
| **Up-regulated genes** | | | |
| APC | +1.12 | 5q21-q22 | **Cell adhesion, inhibits proliferation** |
| BRMS1 | +1.18 | 11q13-q13.2 | **Inhibits metastasis and tumour growth** |
| CCL7 | +1.14 | 16q13 | **Promotes invasion and migration** |
| CDH11 | +1.06 | 16q22.1 | **Involved in the metastatic process** |
| CDKN2A | +1.35 | 9p21 | **Negative regulation of cell cycle** |
| COL4A2 | +1.65 | 13q34 | **The component of extracellular matrix** |
| CST7 | +1.18 | 20p11.21 | **Inhibits cysteine proteinase** |
| CXCL12 | +1.91 | 10q11.1 | **Participates in cell adhesion** |
| EWSR1 | +1.73 | 22q12. | **Transcription factor, promotes oncogenesis** |
| FAT1 | +1.42 | 4q35 | **Participates in cell adhesion** |
| FLT4 | +1.03 | 5q34-q35 | **Promotes tumour metastasis** |
| FXYD5 | +3.00 | 19q12-q13.1 | **Negative regulation of cell adhesion** |
| HRAS | +1.36 | 11p15.5 | **Promotes proliferation** |
| HPRT1 | +1.07 | Xq26.1 | **Nucleotide metabolism** |
| HTATIP2 | +1.01 | 11q13 | **Positively regulates transcription** |
| IGF1 | +1.12 | 12q22-q23 | **Promotes proliferation** |
| IL-1B | +1.45 | 2q14 | **Inhibits or promotes proliferation** |
| KISS1R | +1.56 | 19p13.3 | **Suppresses metastasis** |
| KRAS | +1.15 | 12p12.1 | **Cell signal transduction, proliferation** |
| METAP2 | +1.06 | 12q22 | **Protein hydrolysate and modification** |
| MMP11 | +1.40 | 22q11.23 | **Decomposes, protein hydrolysate**  **and promotes metastasis** |
| MMP2 | +1.67 | 16q13-q21 | **Decomposes, protein hydrolysate and**  **promotes metastasis** |
| MTSS1 | +1.37 | 8p22 | **Inhibits metastasis and proliferation** |
| NME1 | +1.13 | 17q21.3 | **Negatively regulates proliferation**  **and participates in cell adhesion** |
| NME4 | +1.18 | 16p13.3 | **Inhibits proliferation** |
| NR4A3 | +1.59 | 9q22 | **Transcription factor, promotes proliferation** |
| PNN | +1.13 | 14q21.1 | **Inhibits proliferation** |
| RB1 | +1.19 | 13q14.2 | **Negative regulation of cell reproduction** |
| SET | +1.09 | 9q34 | **Inhibits histone acetylate** |
| SMAD2 | +1.21 | 18q21.1 | **Cell signal transduction** |
| SYK | +2.30 | 9q22 | **Promotes proliferation** |
| TNFSF10 | +1.37 | 3q26 | **Induces apoptosis, inhibits proliferation** |
| TRPM1 | +1.21 | 15q13-q14 | **Calcium channels** |
| TSHR | +1.76 | 14q31 | **Promotes proliferation** |
| VEGFA | +1.15 | 6p12 | **Promotes proliferation, metastasis;**  **inhibits apoptosis** |
| **Down-regulated genes** | | | |
| CD44 | -1.53 | 11p13 | **Cell adhesion and stroma attachment** |
| CD82 | -1.76 | 11p11.2 | **Metastasis suppressor** |
| CDH1 | -2.73 | 16q22.1 | **Inhibits tumour metastasis** |
| CDH6 | -1.29 | 5p15.1-p14 | **Osteosis, cell adhesion** |
| CHD4 | -1.16 | 12p13 | **Chromatin assembly and modification** |
| CTBP1 | -1.01 | 4p16 | **Inhibits cell proliferation** |
| CTNNA1 | -1.31 | 5q31 | **Participates in cell adhesion** |
| CTSK | -1.69 | 1q21 | **Protein hydrolysate** |
| CTSL1 | -1.70 | 9q21-q22 | **Protein hydrolysate** |
| CXCR2 | -1.80 | 2q35 | **Mediates** [**neutrophil**](http://en.wikipedia.org/wiki/Neutrophil) **migration** |
| CXCR4 | -1.07 | 2q21 | **Signal transduction, promotes invasion** |
| DENR | -1.04 | 12q24.31 | **Promotes proliferation** |
| EPHB2 | -1.88 | 1p36.1-p35 | **Signal transduction, promotes invasion** |
| ETV4 | -1.27 | 17q21 | **Transcription factor, promotes proliferation** |
| FGFR4 | -1.42 | 5q35.1 | **Promotes invasion** |
| FN1 | -1.27 | 2q34 | **Participates in cell adhesion** |
| GNRH1 | -1.12 | 8p21-p11.2 | **Inhibits cell proliferation** |
| HGF | -1.12 | 7q21.1 | **Participates in proteolysis,**  **promotes proliferation** |
| HPSE | -1.12 | 4q21.3 | **Hydrolyses protein** |
| IL-18 | -1.02 | 11q22.2-q22.3 | **Promotes cell proliferation** |
| ITGA7 | -1.34 | 12q13 | **Participates in cell adhesion** |
| ITGB3 | -1.80 | 17q21.32 | **Participates in cell adhesion** |
| KISS1 | -3.03 | 1q32 | **Suppresses metastasis** |
| MCAM | -2.39 | 11q23.3 | **Participates in cell adhesion** |
| MDM2 | -1.21 | 12q14.3-q15 | **Negative regulation of cell proliferation** |
| MET | -1.29 | 7q31 | **Protooncogene, promotes cell proliferation** |
| MGAT5 | -1.11 | 2q21 | **Promotes metastasis** |
| MMP10 | -1.45 | 11q22.3 | **Protein hydrolysate, promotes metastasis** |
| MMP13 | -1.25 | 11q22.3 | **Decomposes, protein hydrolysate**  **and promotes metastasis** |
| MMP3 | -1.65 | 11q22.3 | **Decomposes, protein hydrolysate**  **and promotes metastasis** |
| MMP7 | -1.27 | 11q21-q22 | **Decomposes, protein hydrolysate**  **and promotes metastasis** |
| MMP9 | -1.25 | 20q11.2-q13.1 | **Decomposes, protein hydrolysate**  **and promotes metastasis** |
| MTA1 | -1.12 | 14q32.3 | **Promotes metastasis** |
| MYC | -1.77 | 8q24.12-24.13 | **Promotes proliferation** |
| MYCL1 | -1.39 | 1p34.2 | **Transcription factor, promotes proliferation** |
| NF2 | -1.89 | 22q12.2 | **Inhibits proliferation** |
| PLAUR | -2.10 | 19q13 | **Activator of plasminogen** |
| PTEN | -1.27 | 10q23.3 | **Inhibits proliferation and metastasis** |
| RORB | -1.65 | 9q22 | **Participates in regulation of transcription** |
| RPL13A | -1.85 | 19q13.3 | **Protein metabolism, promotes proliferation** |
| RPSA | -1.10 | 3p22.2 | **Participates in cell adhesion** |
| SMAD4 | -1.25 | 18q21.1 | **Cell signal transduction** |
| SRC | -1.35 | 20q12-q13 | **Promotes proliferation** |
| SSTR2 | -1.09 | 17q24 | **Inhibits proliferation** |
| TCF20 | -1.36 | 22q13.3 | **Transcription factor** |
| TGFB1 | -1.82 | 19q13.1 | **Inhibits or promotes proliferation,**  **promotes metastasis** |
| TIMP2 | -2.06 | 17q25 | **Inhibits metastasis** |
| TIMP3 | -1.95 | 22q12.3 | **Induces apoptosis, inhibits metastasis** |
| TIMP4 | -1.92 | 3p25 | **Inhibits metastasis** |
| TP53 | -1.09 | 17p13.1 | **Induces apoptosis and cell differentiation** |
